# Achieving Diagnostic Excellence: Roadmaps to Develop and Use Patient-Reported Measures With an Equity Lens

**DOI:** 10.34172/ijhpm.8048

**Published:** 2024-07-06

**Authors:** Kathryn M. McDonald, Kelly T. Gleason, Anushka Jajodia, Helen Haskell, Vadim Dukhanin

**Affiliations:** ^1^Johns Hopkins University School of Nursing, Baltimore, MD, USA.; ^2^Division of General Internal Medicine, Department of Medicine, Johns Hopkins University School of Medicine, Baltimore, MD, USA.; ^3^Mothers Against Medical Error, Columbia, SC, USA.; ^4^Department of Health Policy and Management, Johns Hopkins Bloomberg School of Public Health, Baltimore, MD, USA.

**Keywords:** Diagnostic Errors, Patient Safety, Medical Errors, Human-Centered Design, Patient-Centered

## Abstract

**Background:**

Diagnostic excellence refers to the optimal process to attain an accurate and precise explanation about a patient’s condition and incorporates the perspectives of patients and their care partners. Patient-reported measures (PRMs), designed to capture patient-reported information, have potential to contribute to achieving diagnostic excellence. We aimed to craft a set of roadmaps illustrating goals and guiding the development of PRMs for diagnostic excellence ("Roadmaps").

**Methods:**

We used iterative inputs from environmental literature scans, expert consultations, and patient voice and employed human-centred design (HCD) and equity-focused road-mapping. The culminating activity of these approaches was an Expert Convening.

**Results:**

Use of PRMs can achieve multiple goals for diagnostic excellence, including but not limited to: (1) PRMs for diagnostic continuity, (2) diagnostic PRM alerts, (3) PRM-based quality improvement, (4) PRMs for research, (5) PRMs for routine screening, (6) PRM-based diagnostic excellence population-level patterns, and (7) PRMs supporting patient storytelling. Equity is considered as a cross-cutting goal. Altogether these and future goals support operationalising a vision of patient-reported diagnostic excellence. Roadmaps were developed as a dynamic tool to illustrate PRMs in relation to specific steps with feedback loops to accomplish goals, anticipated timeframes (8-15 years), synergies to foster, and challenges to overcome. Roadmaps are practical in their following PRMs through the stages of development, endorsement, implementation and scaling, and acting upon those measures. Timeframe estimates assume immediate transitions between these stages and no acceleration through incentives and active coordination.

**Conclusion:**

PRMs for diagnostic excellence have potential to connect patient perspectives, equity, and achievable goals. Roadmaps offer a design approach to enable coordinating measurement activities among diverse stakeholders. Roadmaps also highlight versatility in ways patient-reported information can be collected and used, from clinical settings to public health contexts. Patient-reported diagnostic excellence cannot be established as a solely top-down endeavour, but inherently benefits from bottom-up approaches.

## Background

**Key Messages**

**Implications for policy makers**

- Lack of diagnostic excellence, ie, a diagnostic process that is suboptimal and fails to be timely, cost-effective, convenient, and understandable to the patient, has lethal and other harmful consequences that merit focus from the healthcare and public health sectors globally.
- With increasing attention to the diagnostic side of healthcare, especially in health systems that are fragmented or suffering from disparities or low-value care, policy-makers would want to know a set of actionable goals for diagnostic excellence that can be achieved with patient-reported measures (PRMs).
- Developed roadmaps illustrate to policy-makers a strategic vision of the development and implementation of metrics of diagnostic excellence based on patient reporting.

**Implications for the public**

This work on patient-reported diagnostic excellence demonstrates the place and value of patient-reported information, its collection, and its use in improving the process and results of diagnosis for all patients equitably within and outside the healthcare system. Patient voice can be enhanced and transformed into signals that are more operational for health systems and that might support patient representatives and organisations advocating for changes on behalf of other patients. Examples of achieving patient-reported diagnostic excellence include structuring diagnostic storytelling by patients, removing patients’ “labels” tagged through their earlier care experience, and highlighting the importance of focusing on and understanding those who are not engaged or partly engaged with the health system. Diagnostic excellence throughout care is envisioned as co-driven by patients and their care partners.

Diagnostic excellence is the optimal process—timely, cost-effective, convenient, and understandable to the patient—to attain an accurate and precise explanation about a patient’s condition.^1^ Healthcare is increasingly moving from a technocratic, professionally dominated framing of “excellence” to one that seeks and incorporates the values, knowledge, context, actions, and power of patients and their loved ones.^2,3^ For the pursuit of diagnostic excellence, these perspectives of co-production, patient-centredness, and sharing of power between clinicians and patients have deep implications.^3^ Involvement of care partners, such as family, friends, patient advocates, or others who co-manage patient’s care, is equally important.^4,5^ Diagnostic excellence and, more specifically, patient-reported diagnostic excellence should be particularly of interest to health systems across the world that actively involve patient voice and strive for patient engagement, as well as to systems under stress from care fragmentation, disparities in care, and low-value care. Patient reporting, aligned with patient engagement in patient safety, offers a sensible path for diagnostic excellence measurement that relies on information supplied directly from patients or their care partners, but has not yet been charted.

 Literature sources identify four forms of patient-reported information, each with distinctive roles: (1) patient-reported outcomes (PROs), (2) patient-reported experience (PRE) with care, (3) narrative accounts describing encounters with clinicians in a patient’s own words, and (4) complaints or grievances signalling a patient’s distress when treatment or experience falls short of expectations.^6^ Tools that capture the first two forms of information are known as patient-reported outcome measures (PROMs) and patient-reported experience measures (PREMs). We refer to these and other standardised tools designed to measure any forms of patient-reported information as patient-reported measures (PRMs). Ideally, patient reports assess phenomena or constructs for which the patient is the best source of information and that matter most to patients. There is widespread acceptance that patients are the best judge of their own outcomes.^7^ In a new model for diagnostic measurement system, patients and care partners, including via patient reporting, would drive measurement toward meaningful diagnostic outcomes and more open discussion about potential solutions.^8^

 Four primary use cases for PRMs that create value propositions for multiple stakeholders have been described. These include (1) individual patient care decisions, (2) quality assessment and improvement, (3) performance measurement and value-based payment, and (4) population health and research for new evidence to inform clinical practices and guidelines.^9,10^ Real-time monitoring of patient-reported data may help with early identification of problems requiring a prompt response (alerts based on PRMs or PRM alerts), facilitate improved communication between patients and their clinical team, and allow rapid referral to support robust diagnostic processes.^11^ An effective diagnostic measurement system has the following attributes: it has clarity on the purpose of measurement and for whom it is intended; it drives meaningful change in clinical practice and health system design; it can be used to recognise and reward teamwork and shared decision-making between patients and members of the clinical team; and it helps promote accountability on system-level performance for outcomes that matter to patients and clinicians.^8^ To envision a measurement system satisfying these and other attributes, the technique of road-mapping is promising.

 Roadmaps have been used to inform implementation of interventions, more often drawing from and summarising existing experiences, but also allowing for envisioning of scaling and implementation of novel concepts.^12^ Roadmaps can describe specific projects, series of interconnected projects, or system-wide or even across-system endeavours and are often situated within specific time horizons. Longer timeframes and broader scopes require that roadmaps be more dynamic in nature, allowing for course correction and ongoing revisions. Dynamic roadmaps build upon cross-disciplinary collaborations, multi-stakeholder engagement, and diverse funding sources, all of which entail the need for a coordinating entity. Roadmaps often identify actors of the envisioned implementation and document aligned efforts and common challenges. While roadmaps themselves speed up the development process by outlining and allowing users to anticipate actions, they can also provide scripts for steps for implementation, navigating those steps, and avoiding missteps.^12^

 Here, we aim to present both the process of connecting PRMs to multiple goals, and an initial set of illustrative roadmaps for developing feasible, usable, and beneficial PRMs for diagnostic excellence (“Roadmaps”).

## Methods

 For our road-mapping process, we employed equity-focused human-centred design (HCD) and used iterative inputs from environmental literature scans, expert consultations, and patient voice. The culminating activity of all these approaches was the Expert Convening.

###  Environmental Literature Scans

 We created a Framework for Patient-reported Measurement Opportunities of Diagnostic Excellence that charts PRO and PRE domains of diagnostic excellence onto diagnostic journeys of specific patient populations. The set of diagnostic journeys was chosen based on foundational work by the National Quality Forum and contrasted an example description of diagnostic error versus diagnostic excellence for each patient population with the same settings and timing.^13^ The framework summarises both diagnostic error and excellence ends of the continuum, highlighting timing and setting, thereby indicating measurement opportunities for specific diagnostic journey groupings (Figure S1). While developing the framework, the research team’s efforts were informed by the literature on earlier PRM framework development activities and conceptualisations.^7,9,11,14-18^ Other sources were national and international organisations concerned with PRMs, such as the Australian Commission on Safety and Quality in Healthcare, US Department of Health and Human Services’ Patient-Reported Outcomes Measurement Information System (PROMIS), the Organization for Economic Cooperation and Development Patient-Reported Indicators Survey Initiative, Standard Sets of the International Consortium for Health Outcomes Measurement, Patient-Centered Outcomes Research Institute, National Quality Forum, and others. The literature scan identified potential goals for patient-reported diagnostic excellence by referencing both current applications of patient reports in healthcare and suggested applications in the future.^7,13,15^ Those goals were reviewed and refined first by an internal expert group from the project’s institution (Advisory Group) and then finalised at the Expert Convening of an international group. The selection of goals favoured both commonality with general PRMs and uniqueness of diagnostic process.^9,10^ When illustrating goals with measure concepts and use cases, we created analogues to aforementioned patient-reported work using the Framework to focus on aspects particular to diagnostic processes. Similarly, expert input resulted in other literature scans to fill in identified gaps.

###  Expert Consultations 

 The Expert Convening was held virtually in June 2021 as five 2-hour sessions. Each session was offered twice to accommodate attendees across the globe. A total of 24 experts contributed and participated in the Convening. The experts were from institutions located in the United States, Canada, the United Kingdom, Australia, Germany, and Switzerland (See Supplementary file 1). The areas of represented expertise included PRE and PROs, patient advocacy, health services research, program evaluation, healthcare quality and safety, equity and disparities in health, communication, institutional betrayal and integrity, shared decision-making, stakeholder engagement, health economics, health informatics, health policy, implementation science, psychometrics, primary care, and hospital medicine. Experts were chosen based on their relevance to all Roadmap stages from development to acting upon PRMs. The expert group size was a balance between the desired representation and manageability of the group. The experts were identified via literature sources as the authors of the most relevant publications, nominations by the Advisory Group, and referrals from the initially invited experts. The research team consisted of 2 conveyors, 1 HCD expert, a 5-member Advisory Group, and an ad hoc group who piloted the Expert Convening materials.

###  Roadmaps and Their Components

**Figure 1:**
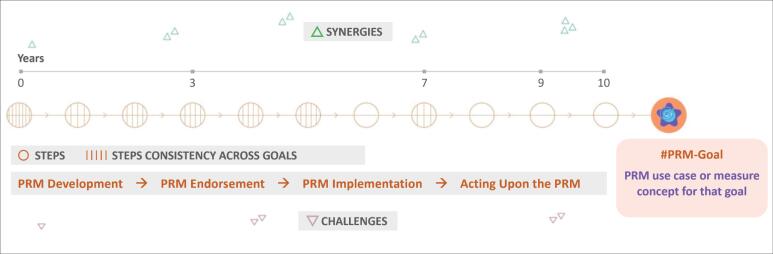
Roadmap Template: Logic and Visual Components. Roadmap usage: road-mapping starts with understanding the goal that diagnostic excellence PRM strives to achieve, then a specific PRM use case or measure concept is used to define a target. Figure elements below are used to envision the PRM development, endorsement, and implementation so that the defined target is met when the PRM is acted upon. Figure elements: #PRM-Goal, diagnostic excellence goal’s hashtag and accompanying pictogram. PRM use case or measure concept for that goal used in defining a target for the generic Roadmap template. Circles depict steps to a PRM use case or measure concept, with the number of perpendicular lines inside each circle representing consistency of that step common across a group of roadmaps targeting different measure concepts or use cases of other goals. Triangles (green and purple) depict synergies and challenges, relative impact on progress (more triangles, more impact), and location relative to associated step. Timeline in calendar years. Abbreviation: PRM, patient-reported measure.

 Roadmaps were designed with visual and logic components that were introduced for expert consultations (Figure 1). A Roadmap features a system-level goal on the far right, as the culmination of PRM stages on the road to that goal: measure development, measure endorsement, measure implementation and scaling, and, finally, acting upon the measure. Steps are depicted for each stage as circles organised linearly, though feedback loops between steps are expected. For integration across a group of roadmaps, the number of perpendicular lines in each circle indicates whether that step is consistent across other goals. For example, when a step is common for seven goals envisioned in this project, it has 7 marks in the step’s circle, while other steps are unique to one envisioned goal (no commonality, no marks). Synergies and challenges are shown above and below the steps, respectively, with their anticipated impact and magnitude (low, moderate, or high) reflected by the number of triangles (one, two, or three, respectively). The location of synergies and challenges symbolises their associations with a specific step in time. Finally, above the line of Roadmap steps is a projected timeline in calendar years. A hashtag convention was recommended by the Advisory Group and applied to provide meaningful linkages between different visual components without any arbitrary numeration or ordering of goals or bullet points.

###  Road-Mapping

 The experts worked in small and large groups during the five sessions to reflect on the materials presented and to critically contribute to road-mapping. The experts additionally contributed via communications with the research team and individual make-up sessions. At session 3, the Convening experts reflected on the correspondence of diagnostic journeys from the Framework with suggested goals by illustratively walking through several examples of patient-reported domains (also see Figure S2). After revisions, the Convening experts selected seven goals as exemplars to demonstrate the versatility of the road-mapping technique for multiple goals. To work on illustrative PRM pathways supporting four of these goals, four expert groups were given a Roadmap draft for refinement. These illustrations were selected as those demonstrating different timelines and directions for PRMs and allowing analysis of common patterns in required steps, synergies, and challenges. The experts were asked to identify missing steps or those requiring clarifications and to identify or modify important synergies and challenges, including ranking their impact as low, moderate, or high. The groups were moderated by research team members trained in HCD and conducting research in diagnostic safety and quality, and the discussions were recorded. At session 4, Roadmaps were presented again to the entire Convening for validation and additional feedback. The experts discussed the timeline for each Roadmap assuming natural development of the field with immediate transitions between the stages. Finally, the Convening weighed in on the consistency of steps and commonalities of synergies and challenges without attempts to standardise those. The Expert Convening participants had opportunities to reflect upon, revisit, refine, or build upon previous decisions that were presented as summaries at each session.

###  Patient Voice

 Throughout this project dedicated to amplifying patient voice via PRMs, we treated patient voice of our participants as a contributor to road-mapping equal to either the expert group or to the literature scans’ input. The patient voice of our work was composed of patient advocates on our internal Advisory Group; patient advocates, leaders, and representatives at the Expert Convening; and facilitated discussions of Roadmaps by patient attendees of the Society to Improve Diagnosis in Medicine’s 14th International Diagnostic Error in Medicine (SIDM 2021) Conference. Those patient advocates themselves or their loved ones experienced harmful diagnostic errors, and now these advocates work with patients across conditions and backgrounds to help them advocate for their health. Additions of new patient-reported domains, modifications to measurement opportunities along diagnostic journeys of patient populations, and revisions of goals of diagnostic excellence PRMs were done based on patient voice. Those representing patient voice were embedded in the research team and had equal control over decision-making at all stages and in all types of decisions.

###  Human- Centred Design with Equity Focus

 HCD is an inclusive and collaborative process approaching members of a community as experts in their own life challenges.^19^ HCD focuses on understanding people in context and from their own perspectives and centres on problem-solving and continuous iterations.^20^ It is a structured and inclusive process that does not rely on a visionary leader, but rather leverages the strengths and insights of the team and community members to increase the likelihood that the solution will be successful.^21^

 HCD guided the process of this work, including iterative methods for interviews with Advisory Group and experts, approaches to composing equitable teams, structure and workflows for the Expert Convening, and active engagement with visualisations. This methodology shaped the co-creation orientation with brainstorming activities, collegial discourse principles, and collaboration among diverse experts to support honest conversations and elicit participant feedback. HCD activities were focused on equity, understood by the team as fairness and justice in our processes and inclusion. For instance, Roadmaps and other illustrations were piloted with internal experts representing various communities (eg, cancer survivors, patients with rare diseases and chronic musculoskeletal conditions) to iteratively enhance Roadmap understandability and to detect patterns that might otherwise go unnoticed.

## Results

**Figure 2:**
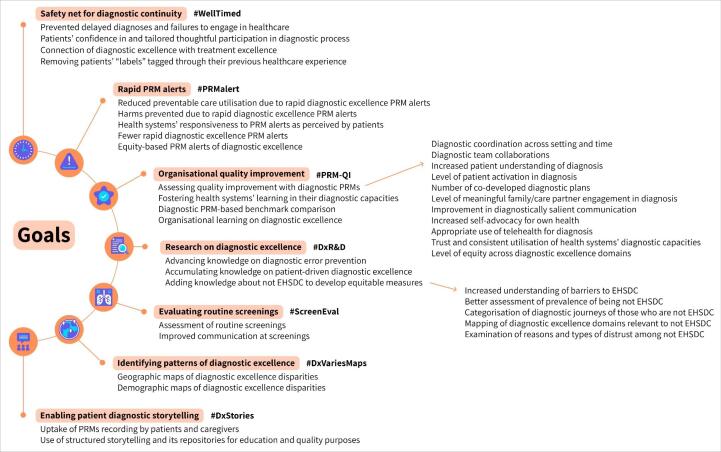
Diagnostic Excellence Goals That Can Be Achieved With Patient-Reported Measures. Abbreviations: EHSDC, engagement with health systems in their diagnostic capacities, including partial engagement; Dx, diagnosis; PRM, patient-reported measure; QI, quality improvement; R&D, research and development. Figure elements: pictorial and #hashtag abbreviations of goals highlighted in orange and bolded; the lists under each goal provide specific examples, including measure concepts (eg, equity-based PRM alerts) and, for some, use cases (eg, geographic maps of diagnostic disparities).

 We present an exemplar set of seven goals that can be achieved for diagnostic excellence by developing and using PRMs (Figure 2 and Figure S7). We highlight the cross-cutting role of health equity and introduce a collective vision of patient-reported diagnostic excellence. Finally, we provide general remarks from the Expert Convening on patient-reported diagnostic excellence and emphasise remarks informed by patient voice.

###  Patient-Reported Diagnostic Excellence Goals and Metrics

Figure 2 presents the seven selected goals; for each goal, we provide examples of measure concepts and use cases that vary in degree of refinement. The seven goals are not exhaustive and complement each other, and those exemplary goals may have overlapping PRMs.

*PRMs for diagnostic continuity (goal 1: #WellTimed). *PRMs can contribute to establishing over time a safety net perceived and reported by patients as experience of having a seamless and connected diagnostic journey and being supported by the health system where necessary. PRMs can be assessed agnostic of setting (and cross-sectionally), but individual patient responses can be tracked for real-time response. As health systems could use these PRMs to support coordination of diagnostic care, prevent lapses in transitions, and identify what disengages people from care, patient reporting could prevent delayed diagnoses and failures to engage or retain people in diagnostic care. Under this goal, PRMs can also be used to monitor patients’ confidence in diagnostic processes and tailor their thoughtful participation in diagnostic processes to help prevent failures to engage or follow-up. Using PRMs can also help with removing patient “labels” tagged through earlier healthcare experiences in their medical records via patients’ assessing that process. Labels such as “difficult,” “noncompliant,” or “malingerer” might introduce biases and cognitive pitfalls in clinicians. As another measure concept example, patients’ experiencing connection of diagnostic excellence with treatment excellence can be measured and achieved.

*Diagnostic PRM alerts (goal 2: #PRMAlert).* PRMs can prompt rapid and real-time alerts aimed at achieving diagnostic excellence. These alerts are collected on an individual basis and require responses from the entity or setting that collected them. For example, patient reports collected after a diagnostic encounter that indicate a patient has not received or understood the explanation for their health concern(s) would trigger a follow-up. The benefit of these alerts can be, for example, the reduction in preventable (unnecessary or duplicative) care utilisation or the prevention of harms. Assessing the responsiveness of health systems to these PRM alerts and the number of these alerts over time, with the assumption that fewer alerts indicate diagnostic excellence, might be other measure concepts. There also should be equity-based PRM alerts of diagnostic excellence aimed at providing proactive responses to diagnostic disparities (eg, an alert for higher rates of immediate diagnostic harms in older adults).

*PRM-based quality improvement (goal 3: #PRM-QI). *PRMs can be used for organisational quality improvement. In this case, PRMs would be collected by individual organisations and aggregated for all patients or subgroups without accountability to respond to a particular patient situation. These PRMs can measure and foster health systems’ learning abouttheir diagnostic capacities, ie, distribution, accessibility, and affordability of their diagnostic resources. These PRMs can serve for diagnostic benchmark comparison that can be further developed into performance measures or quality measures for public quality reporting or value-based payments. To illustrate, the following quality improvements can be assessed by PRMs: diagnostic coordination across setting and time, diagnostic team collaborations, patient understanding of their diagnoses, patient activation in diagnosis, diagnostic plans co-developed with patients, care partner engagement in diagnosis, and improvements in communication so it is salient for diagnosis for both patients and clinicians.

*PRMs for research (goal 4: #DxR*&*D).*PRMs can be used for research on diagnostic excellence, advancing knowledge on diagnostic error prevention, accumulating knowledge on patient-driven diagnostic excellence, or adding knowledge about those patients not engaged (or those who are partly engaged) with the health systems in the system’s diagnostic capacities (EHSDC). An example of engaging patients would be if routine general population screening provides field-tested communication strategies that support patients in following up with other parts of the health system on screening findings. That knowledge on patient engagement will help to further develop equitable measures and can lead—as use case examples specific to those not EHSDC—to increased understanding of barriers, better assessment of prevalence, categorisation of diagnostic journeys, mapping of relevant diagnostic excellence domains, estimation of the patient-reported time and efforts of engaging in the diagnosis, and examination of reasons and types of distrust. It is important to note the acronym EHSDC was chosen as salient internationally and not tied to any country’s healthcare ecosystem (insurance policy, marginalisation, balance of complementary or alternative medicine systems and traditional ones), and the use of “not EHSDC” was suggested by equity experts to not place judgment on those who are partially engaged or not engaged.

*PRMs for routine screening (goal 5: #ScreenEval).* PRMs can help with evaluating routine population screenings that are intended to identify diseases, such as cancer, at the earliest possible stages. Measure concept examples are satisfaction with routine screenings or communication of expectations for screening process, timeline, and how a patient will receive the results.

*PRM-based diagnostic excellence population patterns (goal 6: #DxVariesMaps).*PRMs can be used to identify patterns of diagnostic excellence in different populations if assessed agnostic of setting (cross-sectionally) but in aggregate groups. Such PRMs can contribute, for example, to geographic maps (eg, comparing regions) or demographic maps (eg, comparing populations by income quartiles) of diagnostic excellence disparities.

*PRMs supporting patient storytelling (goal 7: #DxStories).* PRMs can support enabling and structuring patient diagnostic storytelling, where patients share stories of their diagnostic journeys in language and modality of their preference, including using AI technology, and elements of these stories are mapped onto different PRMs. Measure concept examples are: the uptake of PRMs that include the number of recorded stories by patients and care partners and the use of PRM to structure storytelling and index those stories in repositories for education and quality improvement purposes.

 While reviewing numerous goals for PRMs, the experts raised the idea of one aggregate or composite performance measure based on several diagnostic excellence PRMs. Such a composite measure would need to be developed to reflect the goal of equitable patient-centredness as central to a comprehensive assessment of diagnostic excellence. This type of measure could potentially be developed to assess value-based diagnostic care, as another goal example not included in the original set of seven exemplars.

###  Equity Lens for Patient-Reported Diagnostic Excellence 

 The following equity considerations were established as important to guide the development and use of diagnostic excellence PRMs. Diagnostic error disparities are most commonly measured using retrospective data on prevalence of diagnostic errors. In contrast, the equity lens for diagnostic excellence can prospectively consider that disparities occur in numerous instances: (*i*) entrance to or (trustful) engagement with the health system; (*ii*) different previous care experience and vulnerability to being labelled or experiencing discrimination; (*iii*) different use of healthcare diagnostic settings, eg, emergency departments versus specialists; (*iv*) vulnerabilities in communication experience; (*v*) salience and importance of particular diagnostic excellence domains; (*vi*) potentially greater power differential with healthcare providers that influences transparency and honesty in patient reporting; (*vii*) disparities in PRM response rates, including due to the choice of the modality of collection; and (*viii*) disparities in accountability and the health system’s acting upon collected PRMs.

**Figure 3:**
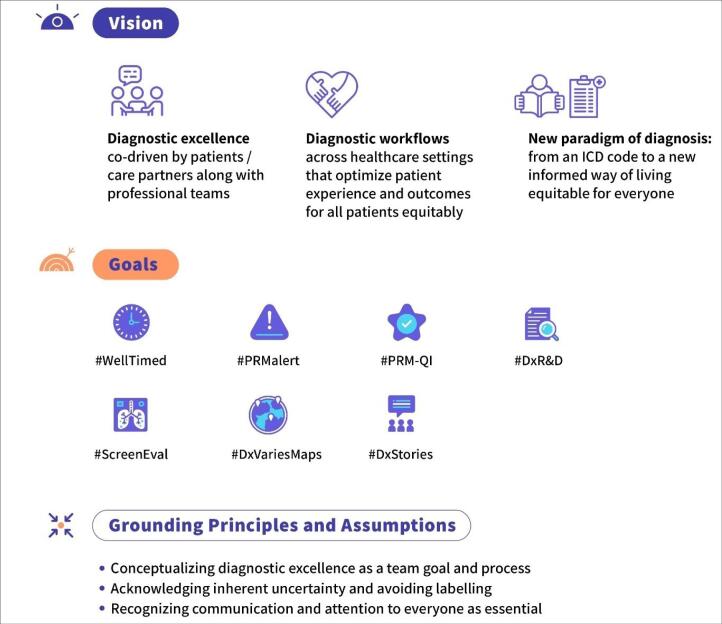
Vision of Patient-Reported Diagnostic Excellence. Hashtags: #WellTimed, safety net for diagnostic continuity using PRMs; #PRMAlert, rapid alerts based on patient reporting of their experience or outcomes of diagnostic excellence; #PRM-QI, PRMs for organisational quality improvement; #DxR&D, PRMs for research on diagnostic excellence; #ScreenEval, PRMs evaluating routine screenings; #DxVariesMaps, identifying patterns of diagnostic excellence with PRMs; #DxStories, PRMs supporting enabling patient diagnostic storytelling. Abbreviations: PRM, patient-reported measure; ICD, International Classification of Diseases; QI, quality improvement.

 Equity was unanimously endorsed as a cross-cutting goal of assessing diagnostic excellence, not as a separate compartment, nor as solely a separate individual measure.^22^ Thus, equity was reflected in the vision of the patient-reported diagnostic excellence (Figure 3). The experts were concerned with the struggle of achieving diagnostic excellence among those who are historically the most disadvantaged. Those who are more likely to encounter diagnostic errors may be the most vulnerable and may have difficulty reporting their experience or outcomes. To illustrate, a PRM might assess level of patient activation in diagnosis with stratification by language, ethnicity, or income level, while another PRM might be specifically assessing experience of discrimination during diagnosis. Diagnostic excellence measurement ought to be sensitive to detect and mitigate (not exacerbate) diagnostic excellence disparities.

###  Vision of Patient-Reported Diagnostic Excellence 

Figure 3 presents a vision of patient-reported diagnostic excellence endorsed by the Expert Convening. First, diagnostic excellence is co-driven by patients/care partners along with professional teams. Second, diagnostic workflows across healthcare settings optimise patient experience and outcomes for all patients equitably. Third, a patient-centred paradigm of diagnosis expands from merely a code from the International Classification of Diseases (ICD) to a holistic and informed way of living, equitable for everyone. This vision is operationalised in the seven exemplar goals discussed above and following grounding principles and assumptions: (1) conceptualising diagnostic excellence as a team goal and process; (2) acknowledging inherent uncertainty and avoiding labelling; and (3) recognising that communication and attention to everyone are essential.

###  Roadmaps Towards Patient-Reported Diagnostic Excellence 

**Figure 4:**
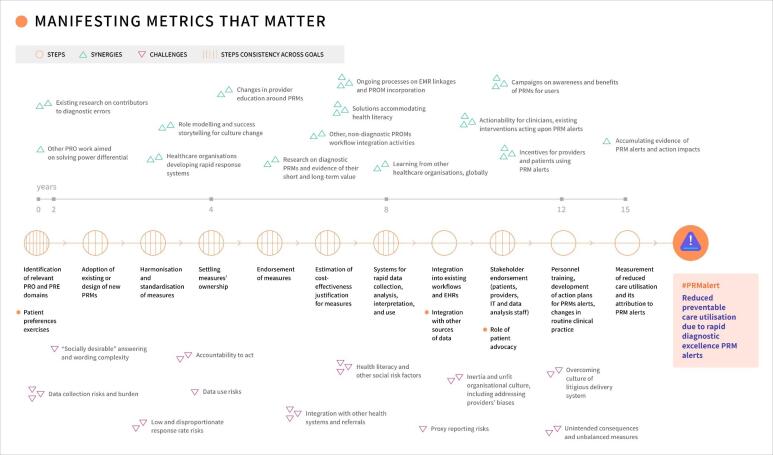
Roadmap Towards Reduced Preventable Care Utilisation Due to Rapid Diagnostic Excellence Patient-Reported Measure Alerts. Abbreviations: PRO, patient-reported outcome; PRE, patient-reported experience; PROM, patient-reported outcome measure; PRM, patient-reported measure; IT, information technology; EHR, electronic health record; EMR, electronic medical record; #PRMalert, rapid diagnostic patient-reported measure-based alerts. Figure elements: #PRMalert - diagnostic excellence goal’s hashtag and accompanying pictogram; reduced preventable care utilisation due to rapid diagnostic excellence PRM alerts - PRM use case for that goal formulated as a target; steps depicted as circles where the number of perpendicular lines inside each circle shows how much that step is consistent across other goals; synergies depicted as green triangles with their impact as low, moderate, or high reflected via the number of triangles; challenges and their magnitude are shown via purple triangles following the same manner; location of synergies and challenges respectively to the step symbolises their association with that specific step in time; timeline in calendar years.

 Four Roadmaps illustrate the development and use of PRMs to achieve patient-reported diagnostic excellence goals. One Roadmap (Figure 4 and Figure S8) is presented here (See Figures S3, S4, and S5 for additional examples). All Roadmaps demonstrate different timelines and directions for PRMs and reveal consistency in required steps, and common patterns in synergies and challenges.

 The Roadmap presented here exemplifies a goal of using rapid diagnostic excellence PRM alerts specifically to reduce preventable care utilisation. Figure 4 displays this Roadmap starting with the steps of the measure development stage (years 0 to 4), specifically: the identification of relevant PRO and PRE domains for the specific goal, adoption of existing or design of new PRMs to measure those domains, harmonisation and standardisation of measures, and settling measures’ ownership (or stewardship). Those steps include feedback loops. Then follows the measure endorsement stage and steps (years 4-8), including estimation of cost-effectiveness as one input for justification of measures. In years 8-12, the measure(s) are implemented and scaled: the systems for rapid PRM data collection, analysis, interpretation, and use are set up; those systems are integrated into existing workflows, into electronic health records, and with other sources of data; then follows stakeholder endorsement for the implementation (by patients, providers, information technology, and data analysis staff) with an active role of patient advocacy. All those steps also include feedback loops. At the final stage, years 12-15, acting upon measure(s), personnel are trained, action plans for responding to PRM alerts are developed, and changes in routine clinical practice are made. This stage implies continuous learning cycles. The timeline ends with the measurement of reduced preventable care utilisation and its attribution to actions triggered by PRM alerts. The Roadmap also delineates synergies and challenges that correspond to the steps on the 0-15-year timeline. Here and elsewhere the timeline assumes no coordinated effort that otherwise might hasten the timeline.

 In comparison, the Roadmap towards improvement in diagnostically salient communication(Figure S3) is an illustration of PRM-based metric efforts exemplifying the organisational quality improvement goal. The timeline also goes through similar steps and feedback loops of the measure development stage, here in 2 versus 4 years, and also follows the measure endorsement stage (year 2). In years 3-4, the PRM is implemented and scaled: the systems for PRM data collection and data aggregation are set up, those systems are integrated into existing workflows, and then follows stakeholder endorsement for the implementation with communication training for providers. At the final stage, years 5-8, acting upon PRM, the analysis of baseline results to design quality improvement activities begins, and then continuous quality improvement activities themselves are conducted and assessed. That timeline ends with the evaluation of improvement in diagnostically salient communication (eg, test results and interpretation) and with actions for this improvement’s dissemination and sustainability.

 The other two Roadmaps cover research on diagnostic excellence (Figure S4), specifically examination of reasons and types of distrust among those not engaged with the health system in their diagnostic capacities, and identification of patterns of diagnostic excellence (Figure S5), specifically production of geographic maps of diagnostic excellence disparities. Those roadmaps have 12- and 10-year timelines, respectively, and follow the same stages of developing the applicable PRM, endorsing the measure, implementing and scaling, and acting upon the measure.

**Figure 5:**
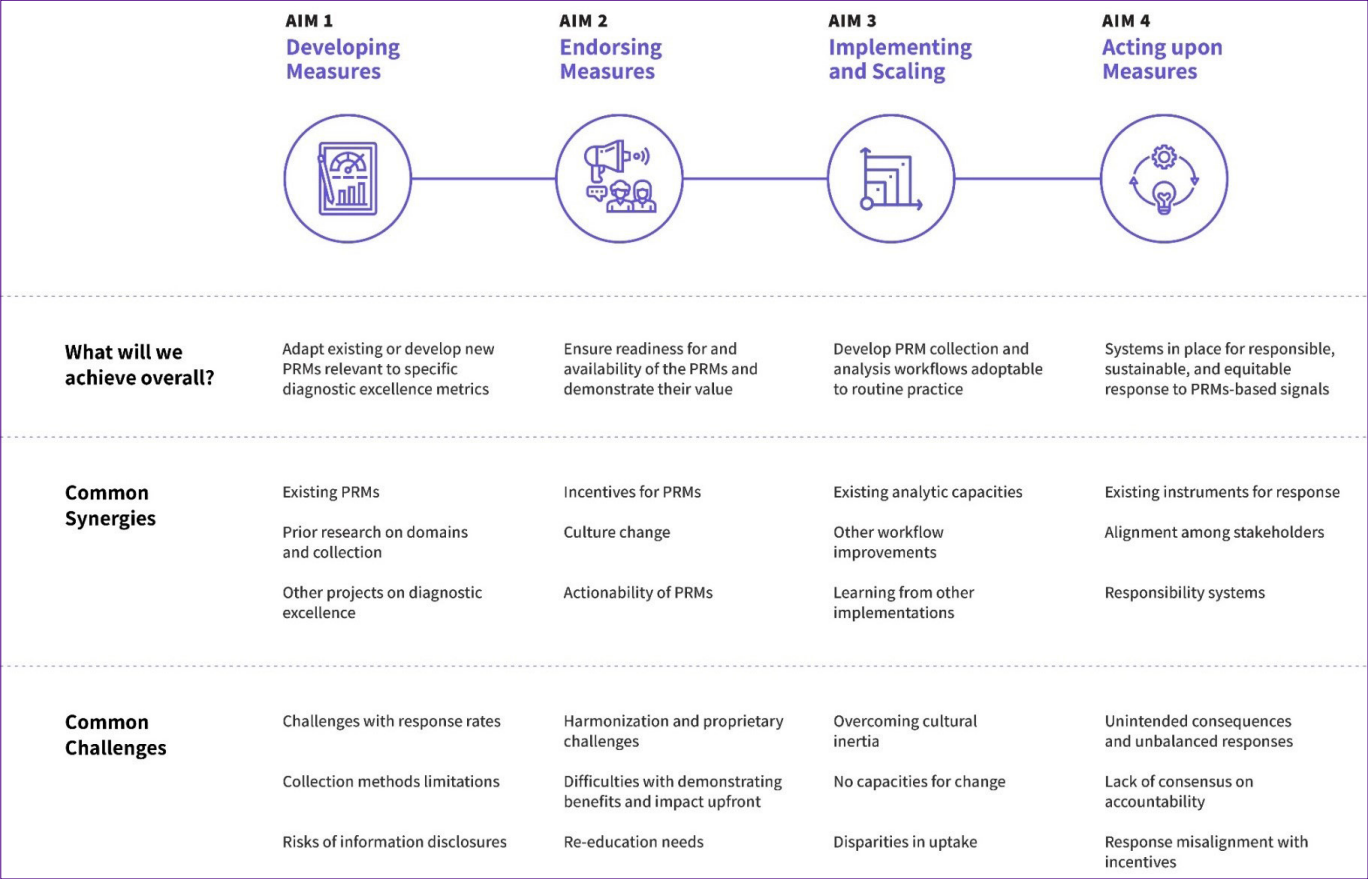
Common Synergies and Challenges Across Roadmaps of Patient-Reported Diagnostic Excellence. Abbreviation: PRM, patient-reported measure.

 The common foundational synergies identified across Roadmaps (Figure 5) during the measure development stage include identifying existing PRMs, prior research on domains and data collection, and other projects on diagnostic excellence. For example, as some healthcare organisations have already implemented rapid response systems, those practices can help with developing PRM-alerts for diagnostic processes (Figure 4). During the measure endorsement stage, the synergies are: incentives for PRMs, culture change around usefulness of PRMs in the diagnostic context, and actionability of PRMs. During measure implementing and scaling, the synergies are: existing analytic capacities, other workflow improvements, and learning from other implementations. For instance, ongoing processes on electronic medical records linkages and PROM incorporation can facilitate setting up systems for PRM-alert data use (Figure 4). Finally, as the measures are being acted upon, the synergies are: existing instruments for response, alignment among stakeholders, and responsibility systems. For example, existing evidence-based communication improvement blueprints can be used as recommended activities responding to issues identified by PRMs of diagnostically salient communication.

 The common initial challenges across Roadmaps (Figure 5) during the measure development stage include: challenges with response rates, collection methods limitations, and risks of information disclosures. During the measure endorsement stage, the challenges are: harmonisation and proprietary challenges, difficulties with demonstrating benefits upfront, and re-education needs. For example, implementing PRM-alerts will face challenges associated with alert fatigue (Figure 4). During measure implementing and scaling, the challenges are: overcoming cultural inertia, no capacities for change, and disparities in uptake. Finally, during the stage of acting upon measures, unintended consequences and unbalanced responses, lack of consensus on accountability, and response misalignment with incentives might become challenges. For example, implementing PRM alerts might face conflicts from a culture of litigious healthcare delivery (Figure 4), where any patient reporting might be perceived as a potential lawsuit as opposed to a learning and improvement opportunity for which reporting would be encouraged.

###  Select Remarks on Patient-Reported Diagnostic Excellence 

 These remarks were chosen to highlight how patient voice brings in public health perspectives and to best exemplify bottom-up directions in road-mapping. Enabling patient diagnostic storytelling and using PRMs to structure storytelling does not have a clear setting, population, or accountable systems stakeholder. However, patient storytelling was prioritised as an important exemplar goal to direct attention in PRM development to explore methods of collecting and standardising patient reports while allowing patients to express their diagnostic outcomes and experience in ways convenient and fulsome for them. Removing patients’ “labels” tagged through earlier patient care experience in their medical records highlights a critical step to avoid propagating inaccurate information and gaps in diagnostic continuity. Such labels could divert attention away from diagnostic accuracy, disengage patients from the diagnostic process, undermine trust, and lead to discriminatory diagnostic experiences. It was noted that only patients can identify and report those “labels,” and the US has no clear health system accountability to respond to reports of problematic “labels.”^23^ Additionally, patient experience of their diagnoses as “labels,” such as a harmless tumour or pre-diabetes, aligns with the broader vision embraced in this project of re-imagining diagnosis in a more patient-centred manner.

 Evaluating routine screening entails dealing with episodic encounters of people within the health system who do not have specific health concerns and might not be otherwise engaged with a given clinical setting. For most of those screened, diagnostic excellence is focused on confirming negative results in a prompt and stress-free manner, but for some, the screening leads to a new diagnosis or situations of experiencing a false positive. A holistic patient-reported diagnostic excellence measurement system needs to embrace a perspective of all those who are screened within or outside of the health system and not limit interest to only those screened positive and who become active patients of a specific medical setting.

 Road-mapping and extensive discussions about applying an equity lens for patient-reported diagnostic excellence highlighted the importance to not only focus on people who are not engaged with the health systems in their diagnostic capacities (and understanding the reasons for that) but also to understand those who are partly engaged. Understanding these populations might expose characteristics of the health system that create vulnerabilities.

###  General Remarks on a Strategy for Patient-Reported Diagnostic Excellence 

 While reviewing Roadmaps, the participants highlighted the commonalities across them, indicating opportunities to synchronise efforts and demonstrate the benefits of assessing diagnostic excellence across several goals simultaneously including by using the same PRMs, if possible. These commonalities might indicate potentials to fast-track roadmap-associated work as opposed to the need for new work and funding.

 The experts advised paying critical attention to early engagement of stakeholders (Figure S6) of patient-reported diagnostic excellence. For instance, an important part that patient advocacy could play is enabling patent-reporting and empowering patient participation.^14^ As PRM-based data can inform and support patient representatives’ advocacy on behalf of other patients; those representatives, such as members of Patient and Family Advisory Councils, would be motivated to have such data routinely collected. Interacting with other stakeholders would require motivating a culture shift around PRMs. Roadmaps would require ongoing interactions with stakeholders to build the capacity to use PRMs and to act upon the measures, as work proceeds through Roadmaps.

 While discussing the patient-reported diagnostic excellence measurement system, the experts were considering the following strategies: (1) building a universal diagnostic excellence measurement system; (2) amassing a diagnostic excellence measurement item bank, scales, and instruments depository; (3) creating shareable computerised algorithms for adaptive administering of diagnostic excellence PRM items; and (4) identifying opportunities for integrating diagnostic excellence PRM items into existing patient-reported measurement systems. Those four strategies were acknowledged as being not necessarily mutually exclusive. They all will require finding additional partners to contribute to an effective measurement system.

## Discussion

 We present a set of exemplary patient-reported diagnostic excellence goals, including illustrative Roadmaps with common synergies and challenges. Roadmaps are dynamic, supporting modification within the scaffolding provided, and facilitating an evolutionary process of learning through inviting constructive criticism, improvements, and revisions by any interested party. Examples of such modifications might be pathways for PRM performance measures for use in public quality reporting and value-based payments,^24^ PRMs that are specific to settings and group of conditions, or PRMs embedded into a learning health system. Roadmaps highlight the promise of PRMs to be particularly valuable for identifying and improving diagnostic disparities.^25^ Roadmaps support strategic progress by highlighting initial challenges and foundational synergies, identifying stakeholders to engage early, and anticipating steps that can be helpful to work on in advance of anticipated 8-15-year (assuming no coordinated effort) timeframes.

###  Strengths and Limitations

 The choice and rigour of the road-mapping methods were motivated by several factors: (1) largely aspirational and future-looking nature of the road-mapped construct and application of analogues; (2) ambitious scope and breadth of the road-mapping approach to tackle real-world settings of national health systems and transcend boundaries; (3) focus on widespread implementation so that achieving exemplary goals serves as feedback on road-mapping; and (4) long-term timeline beyond traditional funding cycles. However, our selection of goals and use cases for Roadmaps did not aim to provide an exhaustive set; thus, some goals are missing. As the role of artificial intelligence (AI) in diagnostic excellence evolves, AI can enhance collection and analysis of patient reporting and accelerate health system’s acting upon PRMs. At the same time, PRMs themselves can inform and humanise AI, another potential goal not considered as an exemplar at the time of Expert Convening.^26^

 Roadmaps were not developed with any specific stakeholder of diagnostic excellence PRMs in mind, including no assumptions about development coming from a top-down (eg, health system) or bottom-up (eg, patients and frontline clinicians) approach. The broad scope of the road-mapping does not focus on specific value propositions of patient-reported diagnostic excellence to specific stakeholders. However, road-mapping does explore bottom-up evaluation of diagnostic excellence, particularly in the context of fragmented health systems or when other gaps for robust diagnostic care delivery exist. In these and other contexts, diagnostic excellence has a high likelihood of emerging bottom-up from patients, care partners, and the people allied with frontline providers rather than simply top-down through policies. In this regard, our vision of patient-reported diagnostic excellence also highlights the gap between the diagnostic process that people want and what they may get, and the benefits of equitable patient-centred grounding principles connected to metrics for diagnostic teamwork and systems accountability. As diagnostic excellence within clinical encounters is envisioned as co-driven by patients and their care partners along with professional teams, diagnostic excellence of the health system relies on patient and public engagement for its co-governance and approaches to measuring what matters. Maintaining a vital equity lens of road-mapping requires the enlargement of inclusivity and additional exposure of this work to diverse groups of patients and the public. That would include a set of patient-facing analogues of this project’s materials that would rely on language and concepts that can engage patients directly.

###  Future Directions

 Roadmaps and exemplary goals delineate the wide range of possibilities for patient-reported diagnostic excellence presenting a solid foundation for this promising measurement-oriented endeavour. This is particularly important at this moment in time when interests of funders and national healthcare quality leaders have increased attention on diagnostic excellence and its assessment.^1,18^ This work demonstrates not only the multiplicity of places and values of PRMs for that effort, but also a path forward. With a few exceptions, minimal systematic and PRM work in the diagnostic space has been reported globally,^27-35^ but those example efforts are growing and align with the paths delineated in Roadmaps. Work on revising Roadmaps should continue and develop new interactions with these important PRM developers and implementors. This will allow Roadmaps to provide a foundation to envision and adopt a holistic and synergistic strategy for patient-reported diagnostic excellence, in addition to all valuable efforts underway. The rationale for developing and demonstrating the road-mapping approach contrasts to chipping away one project at a time and without an eye to the wide implementation that is necessary for meaningful impact for person-centred, diagnostic equity.

 This magnitude of the patient-reported diagnostic excellence endeavour would benefit from a coordinating centre responsible for synchronising efforts of PRM developers and users, updating and further synthesising these Roadmaps, developing additional ones, working with stakeholders, securing hand-offs from research to implementation and scaling, identifying relevant existing expertise, identifying gaps, and aligning funding resources. Roadmaps simply depict the process and immediate transitions between the stages from development to acting upon PRMs and do not by themselves align stakeholder efforts and incentives. To add a coordinated effort, align stakeholders, and hasten the timelines, it requires a strategy akin to the NIH Common Fund initiative, its governing structure, and transcending boundaries, which funded via a multiple-entity mechanism, among others, the Patient-Reported Outcomes Measurement Information System (PROMIS).^36,37^ While many characteristics of patient-reported diagnostic excellence measurement align with PROMIS, it is a unique area that needs a specialised coordinator to tackle lessons learned, support the ambitious area under development, resolve short-term versus long-term goals, facilitate acting upon the PRMs with potential solutions for improving diagnostic excellence, and assure sustainability of the developing measurement system. Maintaining an international vision is also promising, given that the diagnostic excellence community is not restricted to any one country.

 For future immediate steps, working with Roadmaps invites different stakeholders of patient-reported diagnostic excellence (Figure S6) for interactions. For policy-makers, decision-makers, and funders, Roadmaps offer value in presenting potential tools to inform prioritisation and resource allocation. For researchers and practitioners in quality improvement and patient safety, Roadmaps provide value in supporting their future work and goals. For PRM implementers, they add value by showing how existing projects, eg, those awaiting hand-offs to the next stages, are positioned within Roadmaps. For patient advocates and organisations, Roadmaps indicate how patient voice can be enhanced and transformed into signals that are better understandable by the health system and can support patient representatives advocating for changes on behalf of other patients. Convening of stakeholders would ultimately identify natural alliances, actions, and policies to advance patient-reported diagnostic excellence. Those convenings might be localised and specific to stakeholders in specific settings, for example to cancer diagnosis.

## Conclusion

 Patient reporting in the form of PRMs has the potential to provide insights into diagnostic excellence from unique perspectives of patients or their care partners. Roadmaps crafted with an equity lens and HCD expertise offer a design approach to enable coordinating measurement activities among diverse stakeholders through all stages of PRM development and use. If PRMs are either developed or aligned with the road-mapping approach and its further elaborations, patient reporting will be positioned to inform equity aspects of diagnostic excellence proactively. Our work also highlights the need to continue efforts to improve ways to collect patient-reported information and to time that collection, look beyond clinical settings, and incorporate public health perspectives. Patient-reported diagnostic excellence cannot be established only from a top-down approach, but benefits from bottom-up approaches that are inherent to equitable patient reporting.

## Acknowledgements

 We would like to thank our Advisory Group: Mary Catherine Beach, MD, MPH; Norah Crossnohere, PhD; Cyd Eaton, PhD; Richard Skolasky, ScD, MA; Claire Snyder, PhD, MHS; Albert Wu, MD, MPH; Cheryl Dennison Himmelfarb, PhD, RN, ANP, FAAN; and Anna Rappaport, MPH, BSN, RN.

 We are grateful for the technical support from our colleagues: Leta Ashebo, Izzy Rubin, Taharat Sheikh, Owen McManus, Robab Vaziri, Fateha Zannath, Lily Zhu, Jill Williams, Aaron Wiegand, Rishy Peela, Faith Obilo, and Megan Clark.

 Finally, we are immensely grateful to the participants of Expert Convening listed in Supplementary file 1.

## Ethical issues

 Not applicable (no human subjects).

## Competing interests

 Authors declare that they have no competing interests.

## Supplementary files

Supplementary file 1. List of Expert Convening Participants and Supplementary Figures.
