# Supplementary material for: Achieving Diagnostic Excellence: Roadmaps to Develop and Use Patient-Reported Measures With an Equity Lens
Source: Int J Health Policy Manag. 2024 Jul 6;13:8048. doi: 10.34172/ijhpm.8048 (PMC11365170; doi:10.34172/ijhpm.8048)
Supplement: Supplementary file 1 — List of Expert Convening Participants and Supplementary Figures. [file ijhpm-13-8048-s001.pdf]

Article title: Achieving Diagnostic Excellence: Roadmaps to Develop and Use Patient-Reported Measures With an Equity Lens

Journal name: International Journal of Health Policy and Management (IJHPM)

Authors' information: Kathryn M. McDonald[1,2]*, Kelly T. Gleason[1], Anushka Jajodia[1], Helen Haskell[3], Vadim Dukhanin[4]

[1]Johns Hopkins University School of Nursing, Baltimore, MD, USA.

[2]Division of General Internal Medicine, Department of Medicine, Johns Hopkins University School of Medicine, Baltimore, MD, USA.

[3]Mothers Against Medical Error, Columbia, SC, USA.

[4]Department of Health Policy and Management, Johns Hopkins Bloomberg School of Public Health, Baltimore, MD, USA.

***Correspondence to:** Kathryn M. McDonald; Email: kmcdonald@jhu.edu

Citation: McDonald KM, Gleason KT, Jajodia A, Haskell H, Dukhanin V. Achieving diagnostic excellence: roadmaps to develop and use patient-reported measures with an equity lens. Int J Health Policy Manag. 2024;13:8048. doi:10.34172/ijhpm.8048

Supplementary file 1. List of Expert Convening Participants and Supplementary Figures.

Expert Convening Participants

	Participant	Primary Affiliation	Primary Research Interests
**1.**	**Aiyegbusi, Olalekan Lee, MBChB, PhD**	University of Birmingham, UK	Management of chronic conditions, Patient-reported outcomes, Patient and public involvement and engagement
**2.**	**Anhang-Price, Rebecca, PhD**	RAND Corporation, USA	Health Care Quality Measurement, Patient and family care experiences, Program Evaluation
**3.**	**Beaton, Dorcas, PhD**	Institute for Work & Health, Toronto, Canada	Measurement, Translation of measurement into clinical practice, Musculoskeletal disorders
**4.**	**Cleary, Paul D., PhD**	Yale School of Public Health, USA	Methods for using patient reports to improve the quality of medical care, Relationships between clinician and organizational characteristics and the quality of medical care
**5.**	**Dahm, Mary, PhD, MA**	Australian National University, Australia	Communicating for Diagnostic Excellence, Language impact on patient safety and quality of care, Improving critical diagnostic conversations, Managing and communicating uncertainty

	Participant	Primary Affiliation	Primary Research Interests
6.	**Dolka, Io, MS**	Grey Zone, LLC, USA	Patient Advocacy, Patient-Clinician Communication for Diagnostic Excellence, Patient Engagement, Chronic and Rare Medical Conditions
7.	**Etz, Rebecca S., PhD**	Virginia Commonwealth University, USA	Primary care measures, theories of primary care, practice transformation, and stakeholder engagement
8.	**Geraedts, Max, MD,**	Philipps University of Marburg, Germany	Healthcare quality and patient safety, Health Policy, Health Services Research
9.	**Golden, Sara, PhD**	VA Portland Health Care System, USA	Patient-clinician communication, Communication around the risk of cancers, shared decision-making
10.	**Greenhalgh, Joanne, PhD**	University of Leeds, UK	Evaluation of social programs, Healthcare Policy and Practice, Realist methods, Patient Reported Outcome Measures, Clinical decision making
11.	**Hannawa, Annegret F., PhD**	Università della Svizzera italiana, Lugano, Switzerland	Safe communication, error prevention
12.	**Hess, Rachel, MD, MS**	University of Utah Health System, USA	Patient-centered outcomes, Health-related quality of life, Interventions to improve the delivery of health care
13.	**Holve, Erin, PhD**	Department of Health Care Finance, Washington DC Government, USA	Health Policy and Health Services Research
14.	**Langhinrichsen-Rohling, Jennifer, PhD**	University of North Carolina, Charlotte, USA	Community-based research, Underserved and disadvantaged populations, Integration of mental and behavioral health care into primary care and school settings; Institutional betrayal and Institutional integrity
15.	**Lundberg, Brita, MD**	Lundberg Health Advocates, USA	Patient advocacy, Infectious diseases, Diagnostic dilemmas
16.	**Reeve, Bryce, PhD**	Duke University School of Medicine, USA	Patient-reported outcomes, Health-related Quality of Life, Cancer Outcomes, Psychometrics, and Questionnaire design
17.	**Rendle, Katharine A., PhD, MSW, MPH**	Hospital of the University of Pennsylvania, USA	Cancer prevention and care, Healthcare delivery research, Mixed-methods research, Clinical epidemiology, Implementation science
18.	**Repp, Allen B., MD, MSc**	University of Vermont's Larner College of Medicine, USA	Healthcare quality and patient safety, quality measures, Hospital Medicine, General Internal Medicine
19.	**Samuel-Ryals, Cleo A., PhD**	University of North Carolina at Chapel Hill, USA	Disentangling multilevel sources of disparities in palliative and supportive cancer care, Addressing inequities through system-level approaches, Leveraging health informatics tools

	Participant	Primary Affiliation	Primary Research Interests
**20.**	**Sandhu, Alexander, MD, MS**	Stanford Medicine, USA	Health Economics, Implementation of High-value Care Strategies, and Comparative Effectiveness
**21.**	**Schwartz, Bradley L., JD**	Greater National Advocates, USA	Promoting the lifesaving benefits of independent patient advocacy in the clinical setting
**22.**	**Thompson, Cristina J., MS**	University of Wollongong, Australia	Public Health, Health Services Research, Evaluation, Knowledge translation, Implementation science
**23.**	**Valderas, Jose M., MD, PhD, MPH**	University of Exeter Medical School, UK	Patient reports of experiences and outcomes for decision making in clinical practice and health policy, Health care quality and safety, Primary care
**24.**	**Williams, Kate, PhD**	University of Wollongong, Australia	Health and social program evaluation; health outcomes measurement; health services research

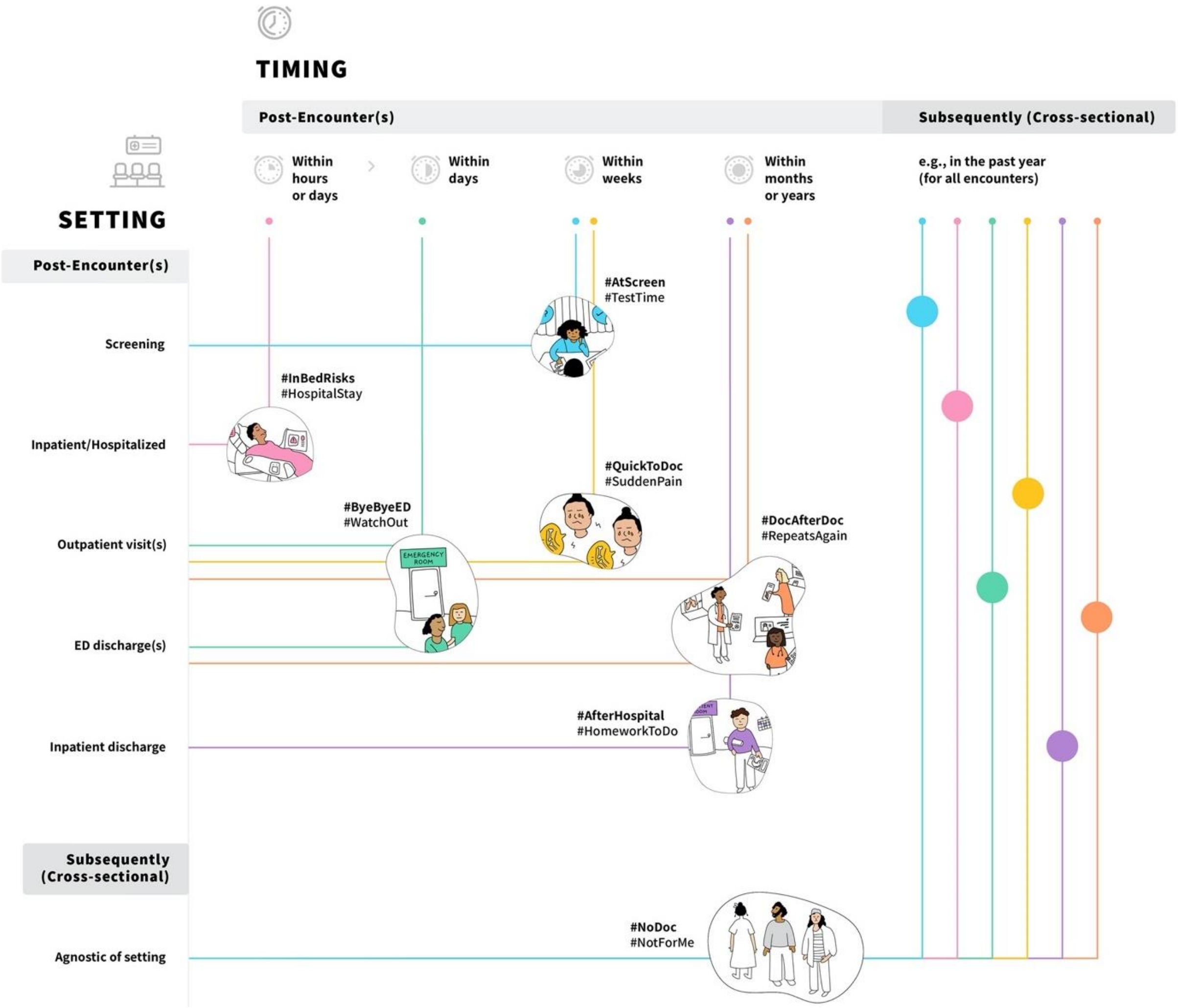

Figure hangtags for diagnostic journey groupings:
#InBedRisks/#HospitalStay – those journeys that occur during hospitalization;
#ByeByeED/#WatchOut – those post emergency care discharge or post an urgent outpatient visit;
#QuickToDoc/#SuddenPain – those post a series of outpatient visits;
#AfterHospital/#HomeworkToDo – those post inpatient discharge;
#DocAfterDoc/#RepeatsAgain – those post multiple outpatient visits or post multiple emergency department visits;
#AtScreen/#TestTime – those post routine screening;
#NoDoc/#NotForMe – those who are not engaged with the health systems in their diagnostic capacities

Figure S1. Timing and setting measurement opportunities. Framework for patient-reported measurement opportunities of diagnostic excellence

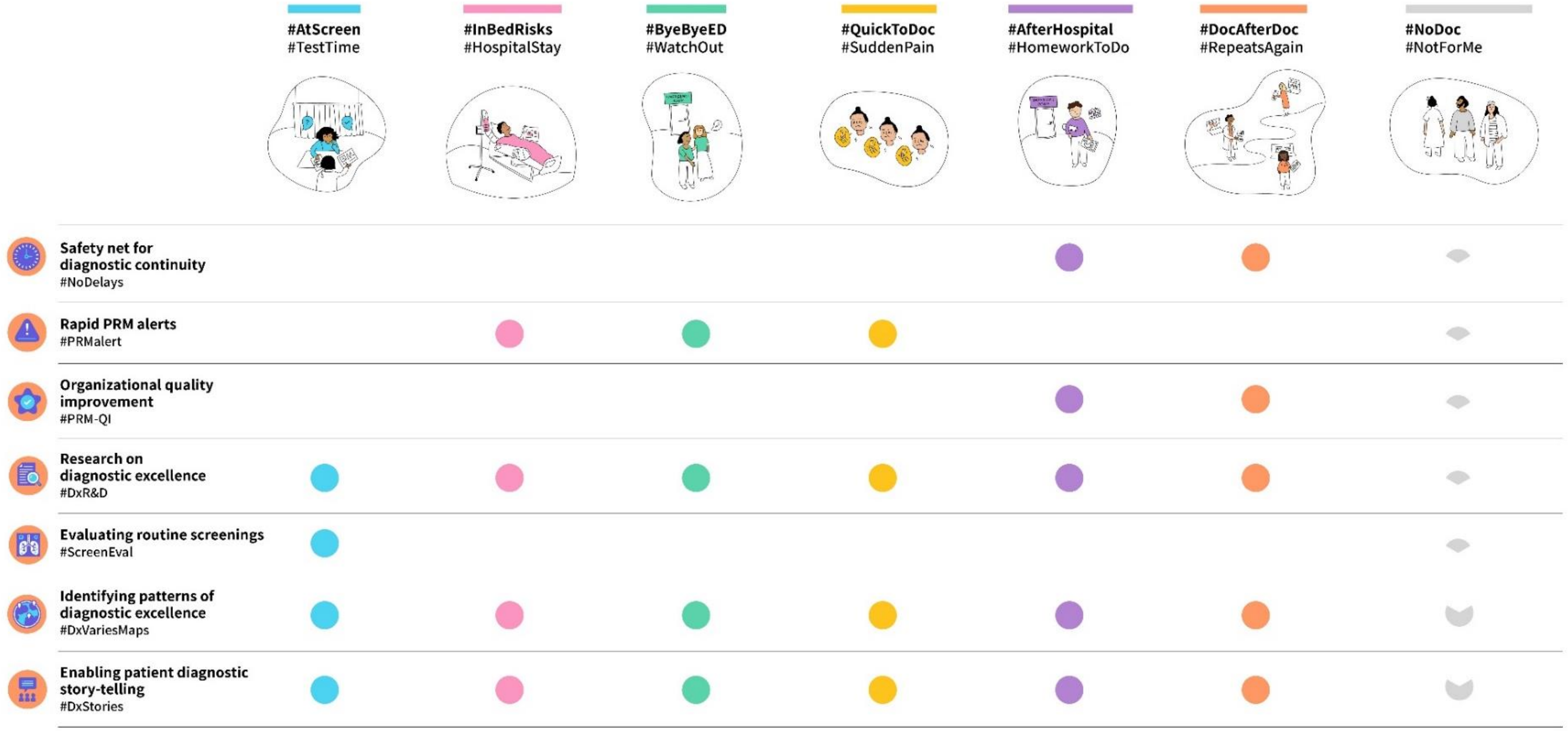

Figure S2. Correspondence of diagnostic excellence goals with Framework's diagnostic journeys

Figure hangtags for diagnostic journey groupings: #InBedRisks/#HospitalStay – those journeys that occur during hospitalization; #ByeByeED/#WatchOut – those post emergency care discharge or post an urgent outpatient visit; #QuickToDoc#SuddenPain - those post a series of outpatient visits; #AfterHospital/#HomeworkToDo – those post inpatient discharge; #DocAfterDoc/#RepeatsAgain – those post multiple outpatient visits or post multiple emergency department visits; #AtScreen/#TestTime - those post routine screening; #NoDoc/#NotForMe – those who are not engaged with the health systems in their diagnostic capacities

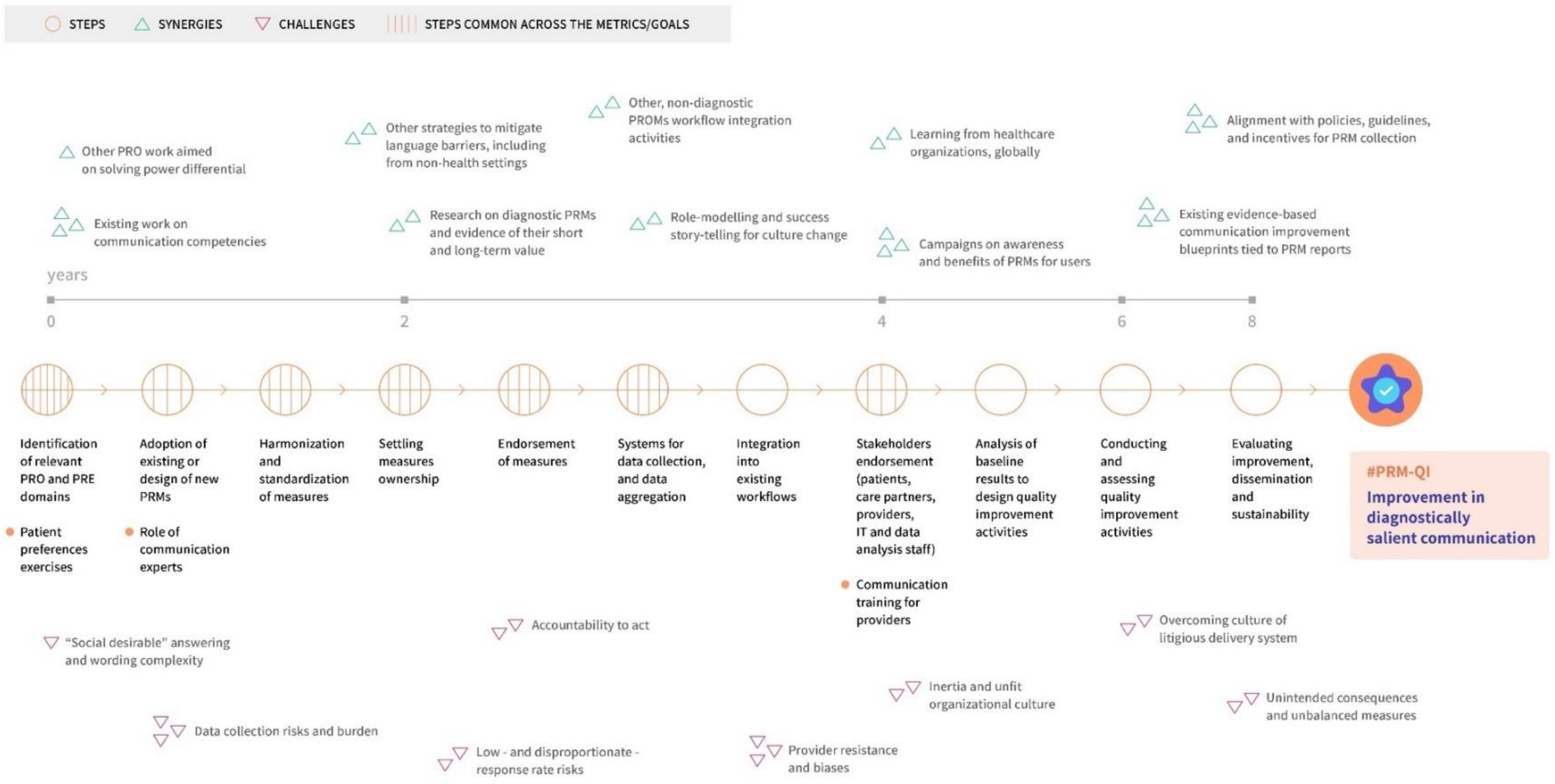

Figure S3. Roadmap towards improvement in diagnostically salient communication

Abbreviations: PRO, patient-reported outcome; PRE, patient-reported experience; PROM, patient-reported outcome measure; PRM, patient-reported measure; IT, information technology; #PRM-QI, PRM-based organizational quality improvement. Figure elements: #PRM-QI - diagnostic excellence goal's hashtag and accompanying pictogram; improvement in diagnostically salient communication - PRM use case for that goal formulated as a target; steps depicted as circles where the number of perpendicular lines inside each circle shows how much that step is consistent across other goals; synergies depicted as green triangles with their impact as low, moderate, or high reflected via the number of triangles; challenges and their magnitude are shown via purple triangles following the same manner; location of synergies and challenges respectively to the step symbolizes their association with that specific step in time; timeline in calendar years.

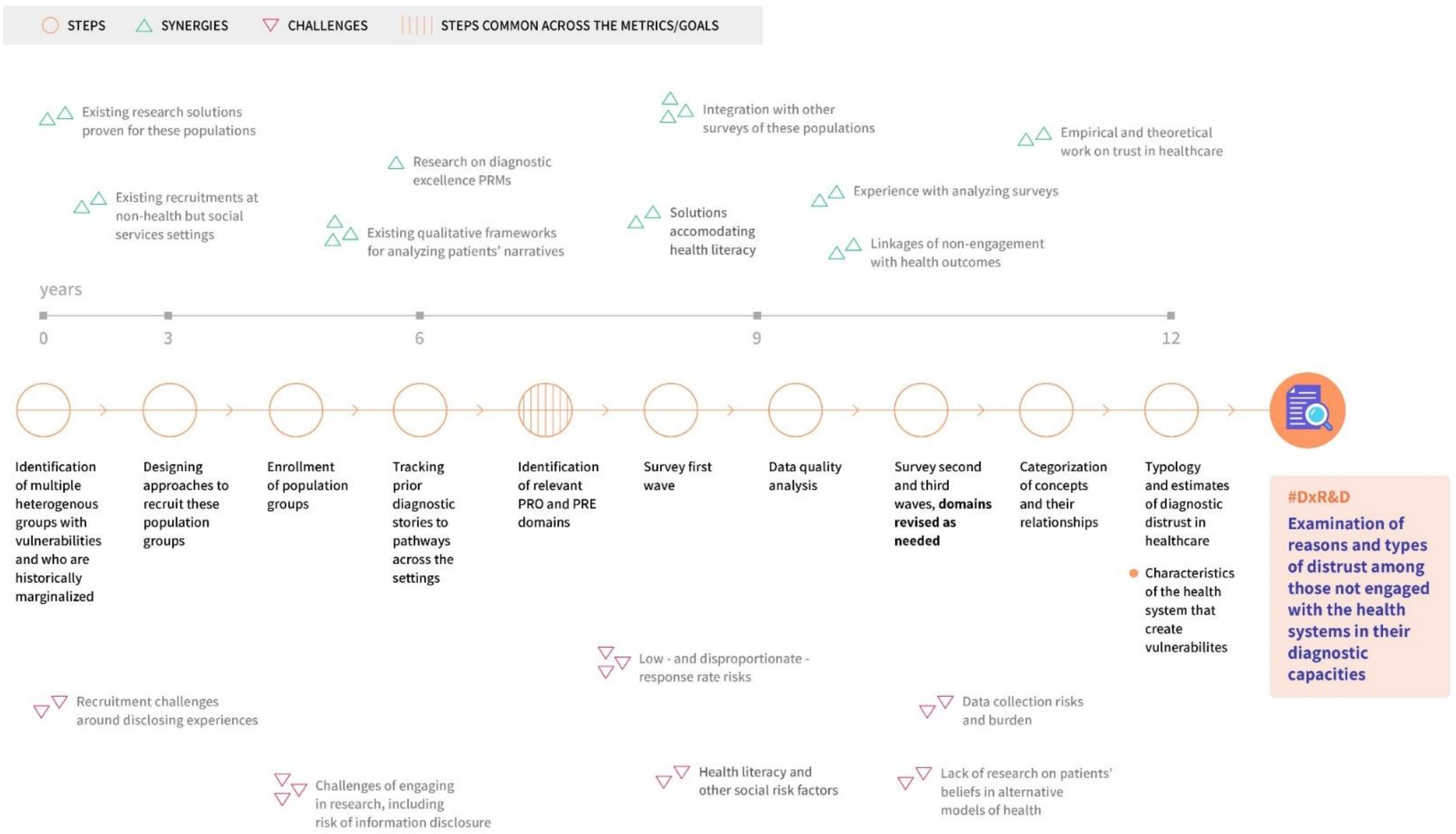

Figure S4. Roadmap towards examination of reasons and types of distrust among those not engaged with the health system in their diagnostic capacities

Abbreviations: PRO, patient-reported outcome; PRE, patient-reported experience; PRM, patient-reported measure as an all-inclusive label; #DxR&D, PRMs for research on diagnostic excellence. Figure elements: #DxR&D - diagnostic excellence goal's hashtag and accompanying pictogram; examination of reasons and types of distrust among those not engaged with the health system in their diagnostic capacities - PRM use case for that goal formulated as a target; steps depicted as circles where the number of perpendicular lines inside each circle shows how much that step is consistent across other goals; synergies depicted as green triangles with their impact as low, moderate, or high reflected via the number of triangles; challenges and their magnitude are shown via purple triangles following the same manner; location of synergies and challenges respectively to the step symbolizes their association with that specific step in time; timeline in calendar years.

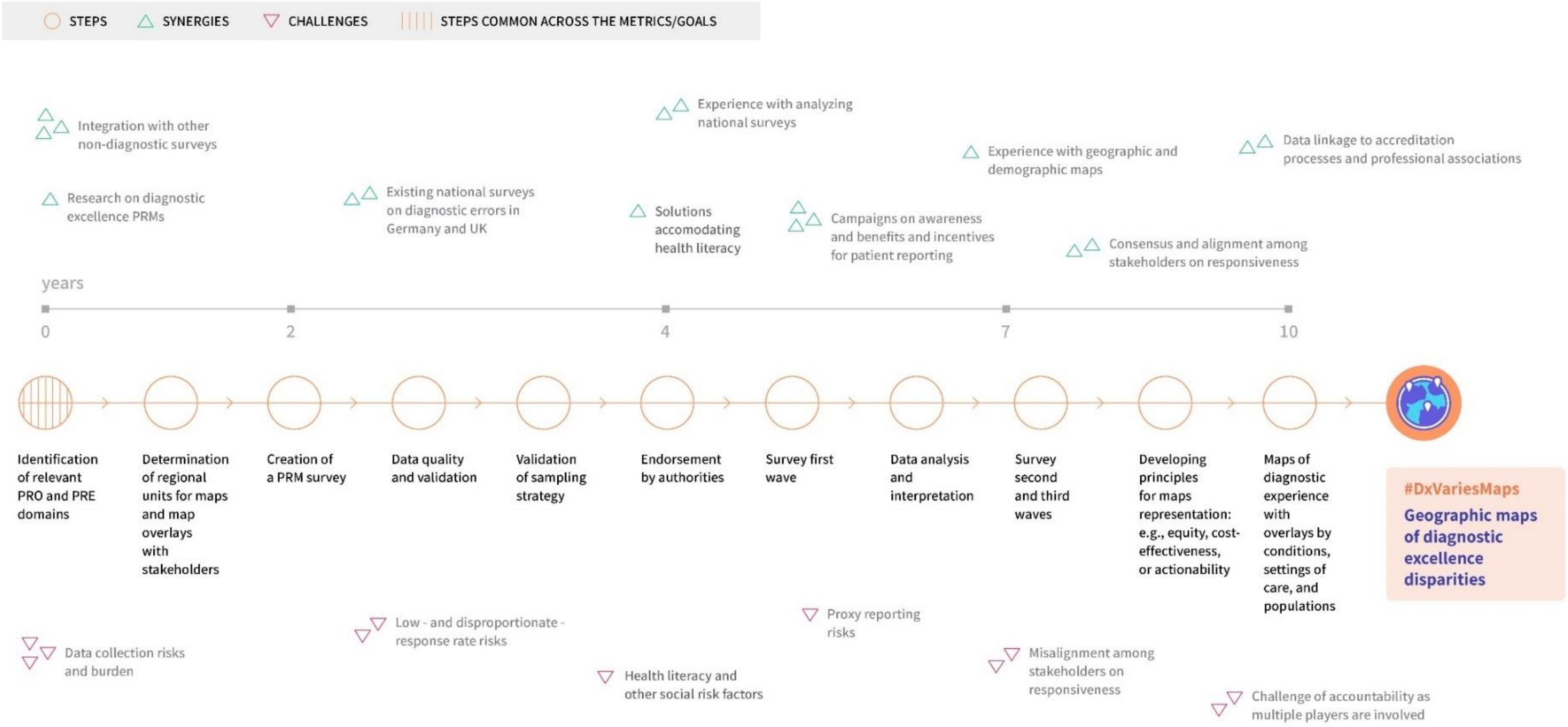

Figure S5. Roadmap towards geographic maps of diagnostic excellence disparities

Abbreviations: PRO, patient-reported outcome; PRE, patient-reported experience; PRM, patient-reported measure as an all-inclusive label; UK, United Kingdom; #DxVaries Maps, identifying patterns of diagnostic excellence with PRMs. Figure elements: #DxVaries Maps - diagnostic excellence goal's hashtag and accompanying pictogram; geographic maps of diagnostic excellence disparities - PRM use case for that goal formulated as a target; steps depicted as circles where the number of perpendicular lines inside each circle shows how much that step is consistent across other goals; synergies depicted as green triangles with their impact as low, moderate, or high reflected via the number of triangles; challenges and their magnitude are shown via purple triangles following the same manner; location of synergies and challenges respectively to the step symbolizes their association with that specific step in time; timeline in calendar years.

Patient-reported diagnostic excellence roadmaps

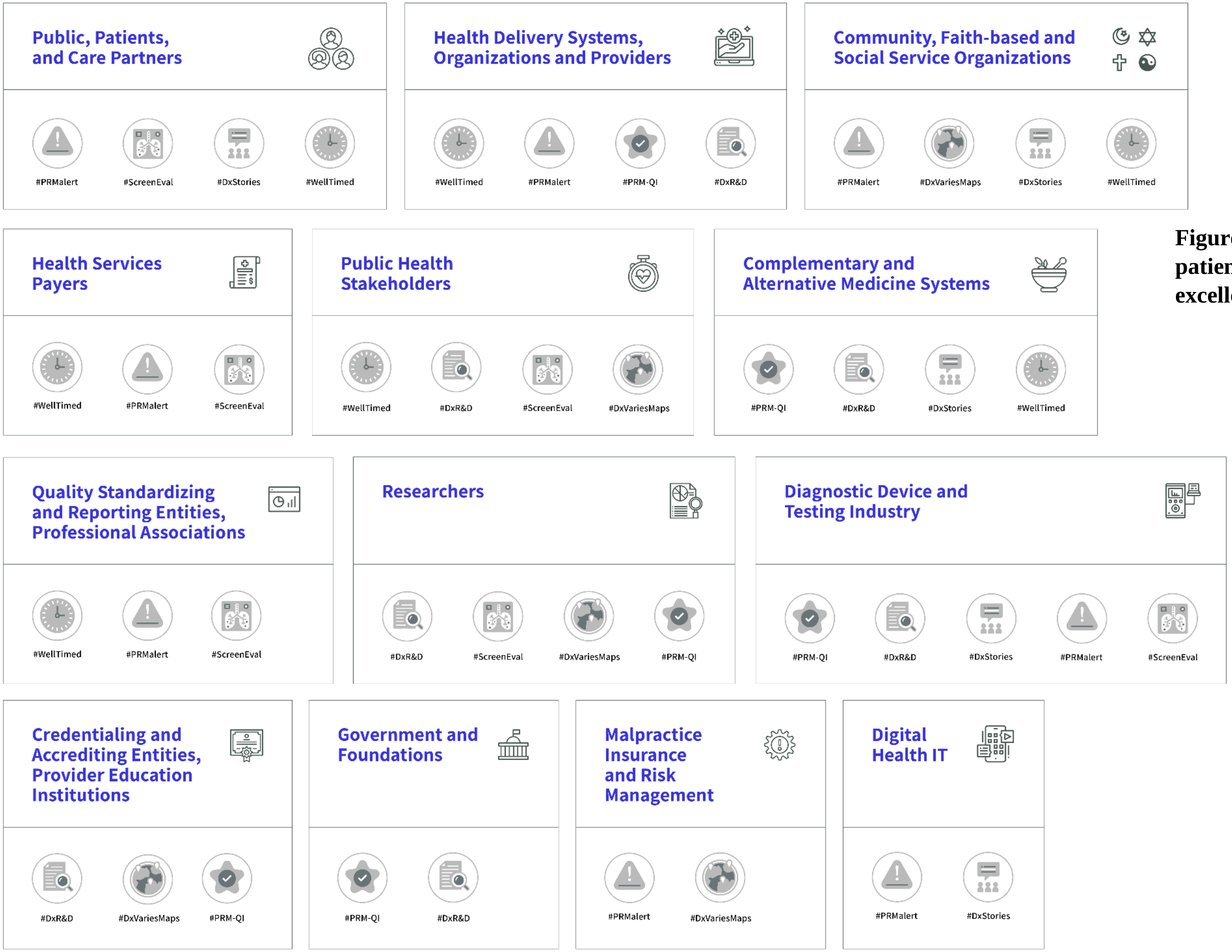

Figure S6. Stakeholders of patient-reported diagnostic excellence

Figure abbreviations/hashtags.

#WellTimed: safety net for diagnostic continuity using PRMs; #PRMAlert: Rapid diagnostic patient-reported measure-based alerts; #PRM-QI: PRMs for organizational quality improvement; #DxR&D: PRMs for research on diagnostic excellence; #ScreenEval: PRMs evaluating routine screenings; #DxVariesMaps: identifying patterns of diagnostic excellence with PRMs; #DxStories: PRMs supporting patient diagnostic story-telling. PRM: patient-reported measure.

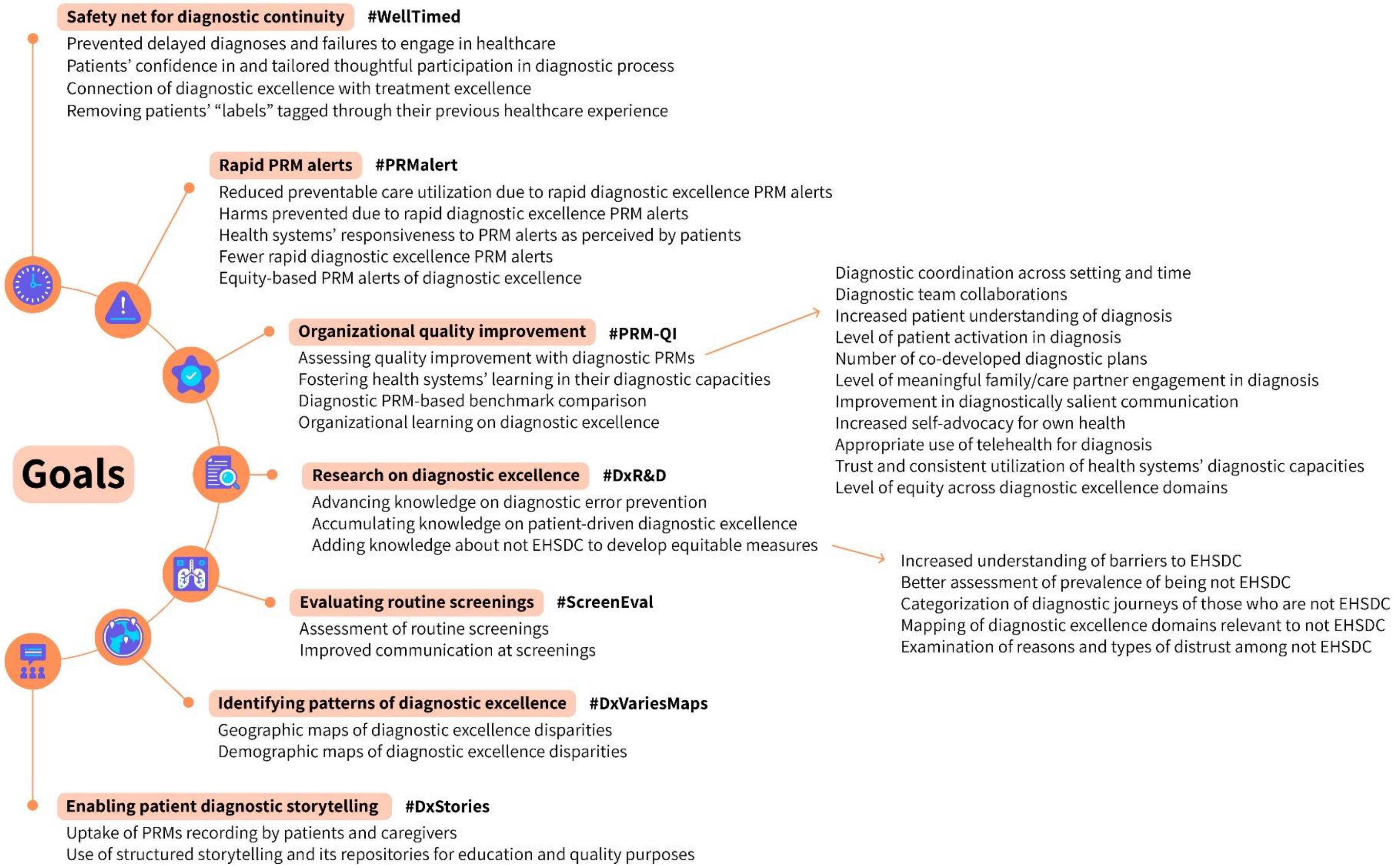

Figure S7. Diagnostic excellence goals that can be achieved with patient-reported measures.

Abbreviations: EHSDC, engagement with health systems in their diagnostic capacities, including partial engagement; Dx, diagnosis; PRM, patient-reported measure; QI, quality improvement; R&D, research and development. Figure elements: pictorial and #hashtag abbreviations of goals highlighted in orange and bolded; the lists under each goal provide specific examples, including measure concepts (e.g., equity-based PRM alerts) and, for some, use cases (e.g., geographic maps of diagnostic disparities).

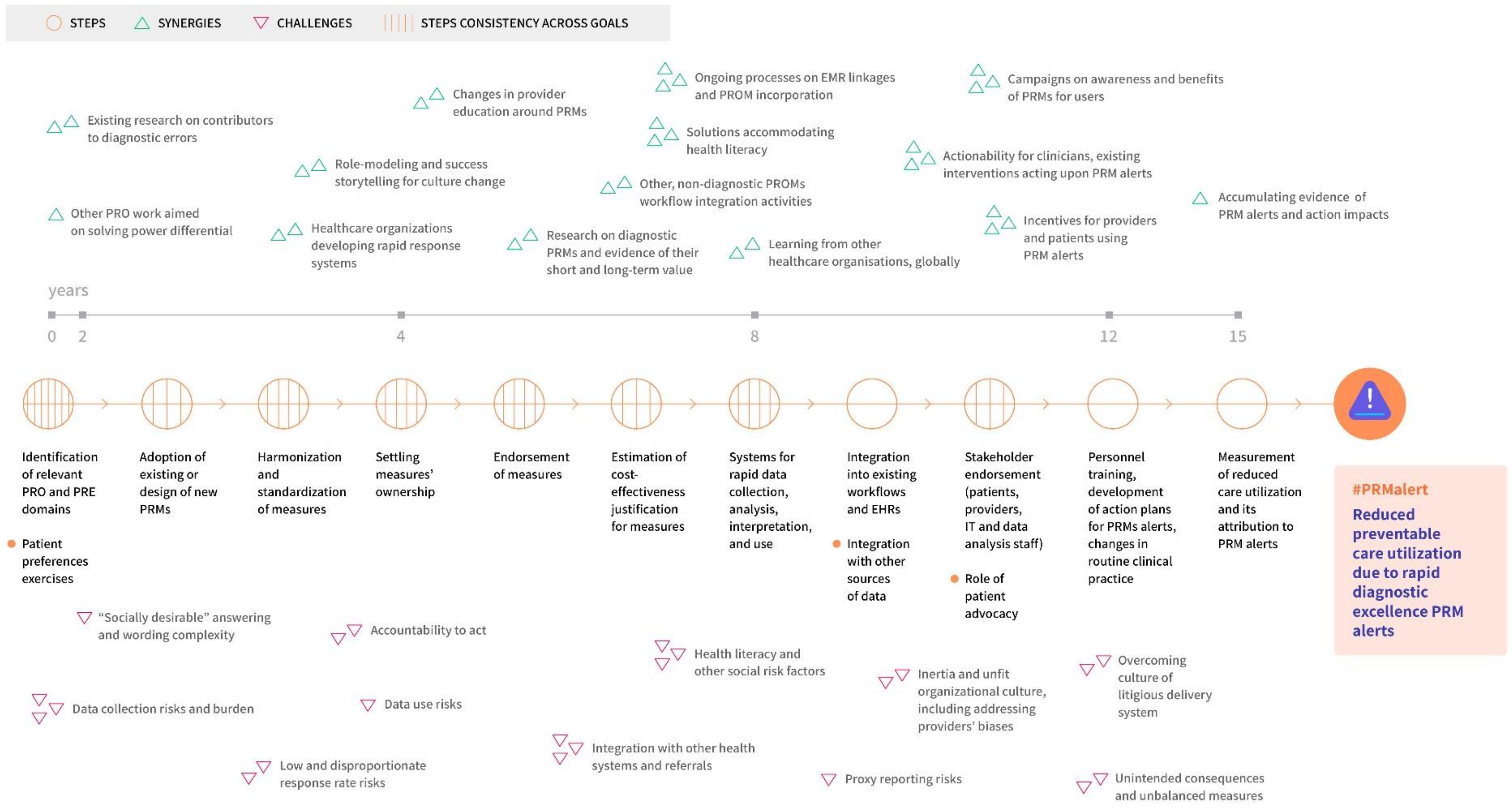

Figure S8. Roadmap towards reduced preventable care utilization due to rapid diagnostic excellence PRM alerts.

Abbreviations: PRO, patient-reported outcome; PRE, patient-reported experience; PROM, patient-reported outcome measure; PRM, patient-reported measure; IT, information technology; EHR, electronic health record; EMR, electronic medical record; #PRMalert, rapid diagnostic patient-reported measure-based alerts. Figure elements: #PRMalert - diagnostic excellence goal's hashtag and accompanying pictogram; reduced preventable care utilization due to rapid diagnostic excellence PRM alerts - PRM use case for that goal formulated as a target; steps depicted as circles where the number of perpendicular lines inside each circle shows how much that step is consistent across other goals; synergies depicted as green triangles with their impact as low, moderate, or high reflected via the number of triangles; challenges and their magnitude are shown via purple triangles following the same manner; location of synergies and challenges respectively to the step symbolises their association with that specific step in time; timeline in calendar years.
